# Exploring the Barriers to Cervical Cancer Screening through the Lens of Implementers and Beneficiaries of the National Screening Program: A Multi-Contextual Study

**DOI:** 10.31557/APJCP.2020.21.8.2209

**Published:** 2020-08

**Authors:** Jyoshma Preema Dsouza, Stephan Van den Broucke, Sanjay Pattanshetty, William Dhoore

**Affiliations:** 1 *School of Public Health, Psychological Research Institute, UCLouvain University of Belgium, Louvain-la-neuve, Belgium. *; 2 *Prasanna School of Public Health, Manipal Academy of Higher Education, Manipal, Karnataka, India. *

**Keywords:** Cervical cancer, screening, prevention, India, low-middle, income countries

## Abstract

**Background:**

Cervical cancer is a major reason for morbidity and mortality in Low and Middle income countries. The National Programme for Prevention and Control of Cancers, Diabetes, Cardiovascular Diseases and Stroke (NPCDCS) sets out broad national guideline to implement Cervical cancer screening. However, an implementation strategy for cervical cancer screening is not in place for districts. Although opportunistic screening takes place, implementation is hindered by psychological and physical barriers for women, as well as insufficient capacity on the part of implementers. This qualitative study aims to identify the specific barriers that prevent the uptake of cervical cancer screening.

**Methods:**

Women who could benefit from cervical cancer program were interviewed to explore the factors that influenced their uptake of the cervical screening offered. Key informant interviews were conducted with implementers of the NPCDCS and with public health staff of three States (Himachal Pradesh, Meghalaya and Karnataka), to understand their perception of determinants of the utilization of screening services.

**Results:**

The general health concern among the participants was low, and routine check-ups were considered unimportant. Poor knowledge about cervical cancer, benefits of screening service availability, as well as a general sense of well-being, embarrassment or anxiety related to the screening procedure, fear of being judged for lack of modesty, and stigma were common barriers to screening uptake. In addition to a general unawareness of cervical cancer geographical inaccessibility of screening as a barrier to participate in cervical cancer screening, in certain regions.

**Conclusion:**

It is essential to increase the knowledge on cervical cancer and on the benefits of screening among Indian women. Providing information and cues to action by health workers and professionals can facilitate the decision to participate. Implementers need to be involved to ensure context specific implementation of the National programme to overcome these barriers.

## Introduction

Cervical cancer is the second most common cancer among women in India. Every year about 96,000 new cases are diagnosed, and about 60,000 women die due to the disease (WHO, 2018). As vaccination against the human papilloma virus (HPV) that causes cervical cancer remains a challenge due to socioeconomic and cultural factors (Sengupta and Nundy, 2005), early detection through screening provides a scope for treatment and better prognosis for the nearly 470 million women in India who are at risk for developing cervical cancer (WHO, 2006). As per recent reports only 22% of the Indian women aged 15-45 years have undergone examination of cervix (NFHS4, 2017). 

To date, there is no nation-wide organised population-based screening programme for cervical cancer screening in India, although facilities for screening are available in private hospitals. However, the latter are only affordable for individuals from middle- or high-income families (Aitken et al., 2013). The facilities for screening are available in District hospitals, but screening is mainly opportunistic, and most of the cases are diagnosed in later stages of development of the disease, with poor prognosis (Mittra et al., 2010). 

In 2010, the Federal Government of India introduced the National Program for prevention and control of Cancer, Diabetes, Cardiovascular Diseases and Stroke (NPCDCS), which included cervical cancer prevention as a comprehensive part. Since its introduction, pilot projects have been undertaken with a view to implement the screening program nation-wide. However, the response to these projects by potential beneficiaries is not high (Sankaranarayanan et al., 2003; Vedantham et al., 2010). Moreover, the factors that influence the uptake of cervical screening by Indian women in the target groups remain largely unexplored. For instance, the operational guidelines of the NPCDCS offer a clear division of tasks. Health promotion, encouraging behaviour change, and the identification of early warning signs of cancer are responsibilities of Primary Health Care Centres (PHCs) located in the peripheries of the country, whereas opportunistic screening needs to be conducted in Community Health Centres (CHCs), which are higher-level health care facilities. Confirmatory diagnosis, treatment and referral must be performed by Tertiary Care hospitals. Yet the pilot projects showed that despite the availability of screening facilities, their utilization depends on various personal, socio-cultural, organisational and health system factors and these factors vary across regions, culture and individuals (Sankaranarayanan et al., 2003; Vedantham et al., 2010). This is in line with the finding that the incidence of cervical cancer itself also shows a variation across different regions of the country (Dhillon et al., 2018), and differs by culture. Hence, in order to improve the uptake of cervical screening in India, the individual, social, economic and cultural factors that may be reasons for poor uptake need to be better understood. 

This qualitative study aimed to explore the individual, social, economic and cultural barriers and facilitators to participate in cervical cancer screening that are experienced by Indian women in the target groups. In addition, it also considers the awareness about these barriers and facilitators among health workers who are tasked with the implementation of the NPCDCS in different contexts of India.

## Materials and Methods

*Study participants*

To reply to the above questions, a qualitative exploratory multi-centric cross-sectional study was performed in three Indian States: Himachal Pradesh, Meghalaya, and Karnataka. Within each State, two to three Districts were selected for the study, and data were collected through focus group discussions and interviews from two distinct groups of participants: 

(1) A first group of participants (hereafter referred to as “beneficiaries”) were women aged 30-59 that could benefit from cervical cancer screening. They were recruited via a convenience sampling method that included waiting rooms from public hospitals, markets, and shops. To be included in the study, participants had to be available for taking part in the data collection and be free of any form of physical or mental illness. 

(2) A second group of participants were State and District Programme managers responsible for the NPCDCS implementation and other health care staff involved in the programme implementation at various levels. They were recruited via a convenience sampling approach. To be included in the study, these key informants had to be directly or indirectly involved in NPCDCS, or in the program implementation or health care delivery for at least 2 years. 

*Data collection*

Women who agreed to participate were contacted by phone or approached by the researcher and, after informed consent was obtained, invited to a Focus Group Discussion (FGD). FGDs were organised until data saturation was obtained. All FGDs were held wherever room was available, in groups of 4-8 participants, and lasted for 20-60 minutes. The group discussions were facilitated by the primary investigator to explored barriers across levels of socio-ecological levels. Women who could not attend the FGDs were interviewed individually. All FGD and interviews were audio recorded with the consent of the participants. A total of six focus group discussions and twelve in-depth interviews were performed. The participants were mostly women from middle- or low-income families, and included fruit sellers, agriculturists, and housewives, but also pharmacists, nurses and health workers. The median age of the participants was 34 yrs. About 80% of the participants had finished secondary education, while 12% had only primary education and few had a bachelor’s degree. The questions of the FGDs and interviews were inspired by the socio-ecological model of health behaviour (Stokols, 1996), and aimed to identify personal, socio-cultural, organisational and health system factors at the individual, interpersonal, community, and public policy levels that could represent barriers or facilitators for cervical cancer screening uptake by beneficiaries. To ensure the credibility of the data collected, quality checks were performed during or at the end of the interviews, and questions were reformulated when necessary. A good rapport was maintained with the participants throughout the FGD or interviews. Open-ended questions were used and context specific clarifications were given to avoid interviewer bias and assure reflexivity.

Interviews with the key informants were held by the primary investigator using a semi-structured interview guide consisting of open-ended, semi-structured questions, based on the socio-ecological model. Interviews were held until data saturation was obtained. Three State and 11 District program managers and coordinators, 7 NPCDCS staff, 9 District gynaecologists and oncologists, 7 Medical officers, a nurse and 6 Health workers were interviewed for the study. State and district Program managers were mostly qualified doctors, while program coordinators were doctors or other health scientists. All the participants had work experience of at least 2 years with NPCDCS program. Health care staff had a minimum of 3-year experience in public health sector. 

*Data management and analysis*

The audio recordings of the focus group discussions and interviews were transcribed by the primary investigator (JD), checked for accuracy by the third author (SP), and translated into English. The translations and quotations made were confirmed by language experts. Directed content thematic analysis (Hsieh and Shannon, 2005) was used to analyse the transcripts of FGD and interviews using Atlas.ti 8 software (Smit, 2002). The barriers to cervical cancer screening uptake thus identified were then categorised across the different levels of the socio-ecological model. For the program implementers, the categorisation was pooled across levels of implementers. Thick descriptions and direct quotations were presented with context specifications to make the data available for use in other settings, thus assuring transferability of the findings. The data analysis was conducted by the primary investigator (JD) for the two groups, and checked for accuracy by two co-investigators (SP and SB) to ensure confirmability. 

*Ethical considerations*

This study is a part of a larger study titled ‘Exploring Health System and Beneficiary Related Determinants of Cervical Cancer Screening Uptake in India’ , the ethical clearance for which was obtained from the Institutional Ethics committee of Kasturba Medical College. Permission was sought from Mission directors of National Health Mission of three states to conduct interviews with program implementers. Written consent was obtained from all participants.

## Results

*Focus group discussions and interviews with beneficiaries Individual barriers to cervical screening uptake*

When asked about their screening behaviour, none of the women who participated in the FGD reported to have undergone screening for cervical cancer in the past. They also generally lacked knowledge on the risk factors and symptoms of cervical cancer. Only five out of the 45 participants (11%) reported to have heard of cervical cancer. These were mostly women with a higher level of education or who worked in the health sector. However, even they were often unsure about the risks of the disease or the need for screening. 

“*What are the signs in the body when one gets cervical cancer*?” (Beneficiary FGD4). 

The FGD participants also seemed unable to distinguish between physiological and pathological changes, which means they could easily miss out on early warning signs for cervical cancer.

 “*Is it ok to get periods a little early like 5 days?*” (Beneficiary FGD4).

A commonly stated reason for this incapability was that ‘*nobody knew about it*’. As such, a lack of knowledge about cervical cancer and about screening seems to be a primary barrier to screening uptake. 

Emotional factors can also be important barriers to screening participation. Some women admitted to be fearful about cancer, and thought that it was better not to know, although for others fear of the disease was actually a facilitator as they consulted a gynaecologist after having observed possible symptoms because they were concerned. Most participants were also unsure about the way the screening procedure was conducted. Fear of pain related to the procedure or embarrassment to undergo screening was a common reason expressed for not participating in screening. Most young women were too shy to discuss the topic. 

A third barrier to screening uptake emerging from the FGD is the low perceived risk. Many women reported that they were too busy with housework, and could not find time to do the test, even when they could afford to be screened in private facilities. The majority of the women also had the feeling they were in good health, and as such did not feel the need to be screened. When those who had no intention to undergo screening were asked for the reasons, the most common reply was that one needs to get screened only when symptoms arise.

“*For those having problems should do this test. Simply otherwise there is no need to do it*” (Beneficiary2)

It was appeared difficult for some of the participants to understand that screening had to be done irrespective of warning signs.

“*I know what are the symptoms. I will tell about it to family and friends, I will tell them to get screened if similar complaints occur*” (Beneficiary10).

Most women were unaware that having had multiple sexual partners is a risk factor for cervical cancer, or felt that they were not susceptible as both they and their husbands were healthy and faithful. Moreover, those who were sexually inactive or had undergone hysterectomy felt less susceptible to develop cervical cancer. There was also some kind of superstitious belief that traditional healers could prevent or treat the disease, which may delay the reporting of cases.

*Interpersonal barriers to cervical screening*

Several women who participated in the FGD reported that their spouses and elders in family were not supportive of them undergoing cervical screening, and were generally unaware of the need for screening. This contributed to women not participating in screening, ignoring the symptoms and reporting only when the symptoms became severe. 

“*Most of the husbands listen to their women. But few don’t…...They will be like ‘you always have same irritating complaints, just keep quiet*” (Beneficiary FGD3). 

 “*If we say we want to do, they will be like ‘Why do you want to do? There is no need. Do you doubt? Have you done something or you think I am not faithful?*” (Beneficiary FGD3) 

The women explained that discussing the topic of screening raised questions about their modesty or trust in their partner, which made them feel uncomfortable to even mention the matter. Whether or not family members supported cervical screening depended on the relationship or understanding between the couple and the health literacy of the family members

*Community barriers to cervical screening*

Religious factors and stigma related to cervical cancer were barriers to screening that were commonly reported by the women in the FGD.

“*Few Muslim women… like for everything they object. Especially when it comes to reproduction.*” (Beneficiary FGD3)

The women explained that there was a stigma related to cervical cancer and that their character and modesty would be questioned if they went for an examinations of their cervix.

Organisational/political barriers to cervical screening 

Participants in the FGD reported that they were not given enough information about the screening facilities that were available in government hospitals and that even health professionals did not recommend screening. 

“*So, I consulted, but did not do any test as I was told that I was fine, sometimes I had itching. I was told it was fungal when there was extensive foul-smelling discharge*” (Beneficiary FGD4) 

Some women felt that long waiting hours in public hospitals discouraged them to participate in screening and to visit hospitals unless it was necessary. On the other hand, free screening services and greater accessibility of the services were considered as potential facilitators to participate in screening. 

*Interviews with implementers*

*Individual barriers to cervical screening uptake*

As revealed by the interviews, the implementers believed that poor literacy and health literacy, financial constraints or poverty, low occupational status and age are the main barriers to cervical cancer screening uptake for women in rural areas. Specifically, most implementers expressed the view that poor knowledge about cervical cancer, its risk factors and symptoms are a vital reason for not undergoing screening. 

“*They should be taught what is normal bleeding and what is pathological bleeding, because they don’t know*” (District Gynaecologist3)

They also stated that beneficiaries have a poor understanding of the need for early detection and of the benefits of cervical cancer screening.

“*Very less patients come at early stages diagnosed. They come with foul smelling discharge, when we do speculum examination and see the growth is always there. If would have screened her earlier, we would have treated her before the cancer goes to advanced stage of CA*” (District oncologist1). 

Implementers also believed that women are negligent about their health status. Most were ‘*busy*’ or just ‘engrossed’ in house work and ‘they don’t come on their own to attend screening, unless they have issues causing discomfort’. ‘*Reluctance*’ or *‘ignorance*’ was often considered to be the reason, along with a perceived sense of well-being among women which made them think that screening was not necessary. In addition, fear related to the disease, the screening procedure or the possibility of a positive diagnosis were also seen as barriers:

“*Women are afraid of the results of the test. If the test comes positive then they may have to worry about expenses of the treatment of the disease. So, they often prefer to not know about their disease status*” (Health worker3)

On the other hand, some implementers thought that fear could also act as a facilitator of screening uptake.

*Interpersonal barriers to cervical screening uptake*

In addition to the individual factors outlined above, a lack of support from the spouse or family members was also mentioned as a barrier to screening participation. Possible reasons for this lack of support, according to the implementers, are poor awareness of cervical cancer risks and inadequate literacy or health literacy on the part of spouses and other family members.

*Socio-cultural and community barriers to cervical screening uptake*

According to the implementers, socio-cultural and community factors including religion, taboo and stigma related to cancer, questions regarding modesty, fear of becoming isolated from one’s family, and lack of freedom to make one’s own decision on health issues can act as barriers to disclose symptoms of cervical cancer and/or to participate in screening. 

‘*Woman can’t go independently anywhere.... she has to take consent from in-laws or husband to come to the hospital*’ (District program manager2).

It was also noted by the health care providers that Muslim women in particular tend to have a poor response towards reproductive health procedures. 

*Organisational and political barriers to cervical screening uptake*

At the organisational level, the implementers believed that the services for cervical cancer screening were inadequate. 

“*People come from far places and when they come here, they don’t get satisfactory things because manpower is very less*” (CHC Nurse1). 

The fact that screening facilities are better equipped in private hospitals as compared to district or government hospitals was perceived as an important inconvenience of the system and a cause of demotivation for women to participate in screening, as women are often referred to other centres. This is especially the case for those residing in remote areas and for poor women living in urban areas.

 “*Even for delivery they are reluctant to come so far because they have to spend for bus expenses*” (ASHA)

Moreover, government hospitals are reportedly crowded and have long waiting hours, which adds to the low accessibility. 

Another barrier that was mentioned are financial constraints. As district hospitals sometimes outsource pathology services that are costly, the cost of PAP test in these hospitals increases. On the other hand, only women with a high level of literacy and a better financial condition can go to private hospitals. 

Implementers also felt that it was insufficient to distribute the handouts and pamphlets on cervical cancer provided through the NPCDCS programme to beneficiaries at health facilities, whilst no active efforts had been put in place to educate the general public. They strongly believed that women were not sufficiently aware of the existing services and that they needed to be made aware of the facilities that are available. Most implementers also believed that women lacked faith in the quality of the services provided by public hospitals, and that there might be a perception that Government health staff is *‘not well qualified to conduct screening*’. Some also assumed that women had lost trust in the health workers themselves.

“*...most of these women don’t come to Government hospitals because they say, that here they put PPIUCD without telling them*” (Health worker3).

Finally, the programme implementers also mentioned the lack of financial incentives as a barrier to the implementation of the NPCDS programme. Despite the existence of several health schemes, there is none that reimburses women for the expenses related to cervical cancer screening.

 The individual, interpersonal, socio-cultural and organisational barriers to participation in cervical cancer screening that emerged from the reports of beneficiaries and implementers are summarized in Table 1.

**Table 1:** Barriers to Cervical Cancer Screening Uptake in India Across Socio-Ecological Levels

Individual barriers	Interpersonal barriers	Socio-cultural and community barriers	Organizational and Political
Demographic factors	Spouse	Religion, Stigma and fear regarding cancer	Insufficient availability of screening services
Age, education, socio-economic status	-Lack of interest		
Cognitive factors	-Low literacy	Gender roles	Inaccessibility
-Poor knowledge of the disease symptoms, process, and risk factors	-Poor knowledge and awareness of cervical cancer and screening		-Geographical and structural
-Poor knowledge of screening	-Low health literacy	Emphasis on modesty	-Financial
-Low health literacy	-Relationship status		-Cognitive
Perceived risk	Family members		
-Underestimation of the personal vulnerability for cervical cancer	-Low literacy		Insufficient information about (availability of) screening services
-Underestimation of the disease consequences	-Poor health literacy		
Emotional factors	-Poor knowledge and awareness		
-Fear and anxiety (related to the disease, diagnosis, expenses if diagnosed, pain related to procedure)	-Superstitious beliefs		
-Shame and embarrassment			
-Superstitious beliefs, stigma			

## Discussion

This study explored the factors that influence women in India to participate in cervical cancer screening. Due to its qualitative nature and the fact that the participants were recruited via convenience sampling the findings of the study cannot be generalised to the population. Nevertheless, the focus group discussions and interviews held with both beneficiaries and implementers allowed us to access in-depth knowledge regarding factors at various levels that, as perceived by those involved, act as barriers or facilitators for cervical cancer screening uptake in India. 

Our findings revealed that women don’t participate in screening for a variety of individual, interpersonal, socio-cultural and organisational reasons. Apart from a general lack of knowledge about cervical cancer, the possible benefits of screening, and the availability of screening services, there also seems to be a low level of perceived risk among women in the target groups, as well as emotional barriers to participation in screening. The latter include fear about cancer, uncertainty and embarrassment about the screening procedure, fear of pain caused by the screening procedure and shyness to discuss the topic. This is further complicated by the overall feeling that spouses and family elders are generally not supportive of cervical screening, and that religious beliefs and stigma also act against participating in screening. Participants in the study thought that the information provided by the government and about health professionals about cervical cancer and about the screening facilities that are available are not sufficient. 

These barriers to cervical cancer screening are also recognized by the implementers of the NPCDS, especially by health workers who implement the program locally. Whilst program managers focused mostly on barriers within the health system as reasons for low screening uptake, implementers (mostly nurses) were aware of most of the cognitive, emotional and interpersonal barriers expressed by the beneficiaries. 

It should be noted that although there was not much difference between the barriers identified by women in the three participating States, women and implementers in the States of Himachal Pradesh and Meghalaya and rural Karnataka in particular also mentioned the geographical difficulty to access health care services, and thought that participation in screening was made difficult by the fact that screening services are only available in selected facilities. Furthermore, there is also an effect of socio-economic level, in the sense that working women seemed to have a more positive attitude towards screening and a higher intention to get screened, as they perceived fewer socio-cultural barriers.

To address the abovementioned barriers with a view to enhance screening uptake, different measures can be taken. At the organisational level, the health system can be strengthened by making more and better screening services available at community health centres, since accessibility to health services is known to improve screening uptake (Lyimo and Beran, 2012). Previous studies have demonstrated that health system factors such as poor quality of services, long waiting hours, or poor response of health care providers leading to a lack of trust in health care staff are important for screening uptake (Singh and Badaya, 2012; Mohanan et al., 2016). This study confirms that health care staff are not proactive or keen to conduct cervical cancer screening. Many health professionals had not undergone screening themselves, and may hold personal beliefs that are against screening (Shah et al., 2012). Supplying adequate manpower and logistics can reduce waiting hours, while training health professionals to deliver patient centred care, monitoring and supervision of health professionals and activities, and involving health counsellors to gain informed consent can help to improve the quality of services and help individuals to gain trust in the health system. 

In addition, making the screening services available for minimal/ no cost or reimbursing the costs through health schemes would reduce the financial barrier to screening uptake. Although this was not addressed in this study, a lack of accessibility may be particularly relevant for women in rural areas or in particular vulnerable groups, such as sex workers (Devarapalli et al., 2018). Therefore, interventions targeting women from rural areas or specific vulnerable groups would be an asset. 

At the individual level, better and more targeted information should be provided to address the general lack of knowledge that was recognized as an important barrier. A lack of adequate knowledge about the disease process or the benefits of screening not only prevents women from initiating the decision-making process towards participating in screening (Lyimo and Beran, 2012), but might also lead to fear for the disease or for the screening procedure (Aswathy et al., 2012; Dahiya et al., 2019). Like other studies (Darj et al., 2019), ours revealed that there are no active efforts to increase screening uptake, and that routine health check-ups are given a low priority, leading to the situation where screening is only done when symptoms are present (Markovic et al., 2005; Wong et al., 2008). Involving ASHA s, ANMs , NGOs and community groups in health promotion and using multiple approaches to disseminate information about screening could be used to help deliver culturally acceptable messages to influence their attitude and change beliefs related to disease and screening (Pirzadeh and Mazaheri., 2012); Krok-Schoen et al., 2016). It is important to note that providing information in itself will not be sufficient to improve screening uptake. As outlined in Health Behaviour Theories (HBT), the decision to perform a behaviour (such as participating in cancer screening) is not a rational choice that depends on objective information, but is influenced by various behavioural, social and self-efficacy beliefs (Marston and King, 2006). It would therefore be recommended to apply HBT to understand the factors when designing intervention to increase screening uptake.

The literacy, health literacy, and beliefs of women about cervical cancer are strongly influenced by their social and cultural environment. The beliefs and attitudes of spouses and family with regard to cervical screening was found to be an important factor in screening uptake in our study, as was also the case in previous research (Devarapalli et al., 2018). Hence, CBPR needs to be considered to involve families and community members to improve their knowledge (Nguyen et al., 2006), build awareness, reduce stigma and superstitious beliefs (Agurto et al., 2004) and emphasize the benefits of early detection of cervical cancer. The involvement of religious leaders may reduce religion related barriers that could also influence screening uptake (Vahabi and Lofters, 2016). Culturally appropriate health promotion measures, aimed at raising the awareness of the need for screening irrespective of the presence of warning signs or the person’s literacy, employment status or socio-economic background can help to increase the utilization of these services. Involving all cadres of the health system staff as well as representatives of the beneficiaries in the implementation planning may allow the development of context specific implementation and ensure a better utilization of the existing facilities.
